# Gene Expression Analysis Suggests Bone Development-Related Genes GDF5 and DIO2 Are Involved in the Development of Kashin-Beck Disease in Children Rather than Adults

**DOI:** 10.1371/journal.pone.0103618

**Published:** 2014-07-29

**Authors:** Yan Wen, Feng Zhang, Chunyan Li, Shulan He, Wuhong Tan, Yanxia Lei, Qiang Zhang, Hanjie Yu, Jingjing Zheng, Xiong Guo

**Affiliations:** 1 School of Public Health, Health Science Center, Key Laboratory of Environment and Gene Related Diseases of Ministry Education, Key Laboratory of Trace Elements and Endemic Diseases of Ministry Health, Xi'an Jiaotong University, Xi'an, Shaanxi, P. R. China; 2 Department of Kashin-Beck Diseases, Qinghai Institute for Endemic Disease Control and Prevention, Xining, Qinghai, P. R. China; 3 National Engineering Research Center for Miniaturized Detection Systems, Northwest University, Xi'an, Shaanxi, P. R. China; University of Maryland School of Medicine, United States of America

## Abstract

**Objective:**

To investigate the differences in gene expression between children and adults with Kashin-Beck disease (KBD).

**Methods:**

12 children with KBD and 12 healthy children were selected and divided into 4 KBD vs. control pairs matched according to age and gender, with each pair having 3 KBD children and 3 healthy children. Additionally, 15 adults with KBD and 15 healthy adults were selected and divided into 5 KBD vs. control pairs matched according to age and gender, with each pair having 3 KBD adults and 3 healthy adults. Total RNA was isolated from peripheral blood mononuclear cells (PBMCs) respectively. A total of 367 target genes were selected based on previous genome-wide gene expression profile analysis. Expression levels of the 367 genes were evaluated by customized oligonucleotide microarray and the differentially expressed genes were identified. Quantitative real-time reverse transcription polymerase chain reaction (qRT-PCR) was conducted to validate the microarray data.

**Results:**

A total of 95 (25.9%) genes in KBD children and 158 (43.1%) genes in KBD adults were found to exhibit more than two-fold change in gene expression level relative to healthy controls. By comparing differentially expressed genes identified in KBD children to those of KBD adults, 42 genes were found to be differentially expressed only in KBD children. And 105 genes were found to be differentially expressed only in KBD adults. Further, 16 differentially expressed genes common to both KBD children and adults were found to be asynchronously expressed in KBD children compared to KBD adults.

**Conclusion:**

Significant differences in gene expression pattern were identified between KBD children and KBD adults, indicating different molecular mechanisms underlying cartilage lesions of KBD children and KBD adults. In addition, bone development-related genes GDF5 (expression ratio = 2.14±0.02) and DIO2 (expression ratio = 0.11±0.05) may contribute to the development of KBD in children rather than in adults.

## Introduction

Kashin-Beck disease (KBD) is an endemic and chronic osteochondropathy with unknown etiology. The disease mostly occurs in children between the ages of 3 and 13 in a diagonal belt-like area ranging from Northeast to Southwest China, with no observed difference in occurrence between genders. Few new KBD patients are observed among adolescents and adults [1]. It has been reported that more than 2.5 million people in China suffer from KBD and about 30 million people are at risk. The prevalence of KBD in children aged from 7 to 13 years reached 50% in serious KBD areas [2].

KBD is characterized by focal chondronecrosis in the mature chondrocytes of articular cartilage and the growth plate cartilage, which can result in impaired endochondral ossification during childhood [3]. Moreover, the premature closure of the epiphyseal plate in KBD children leads to irregular and impaired skeletal development. Enlarged and deformed joints as well as shortened long bones resulting from irreversible bone dysplasia occur during growth. Secondary osteoarthritis becomes the primary clinical manifestation of adult KBD patients.

Compared to adult KBD patients, abnormal radiological signs in the metaphyseal area and retarded skeletal development are more representative in child KBD patients [4]. Therefore, child KBD samples should provide more useful information regarding the mechanism of primary cartilage lesions and enable development of effective prevention and treatment measures for KBD. However, due to the difficulty in collecting child KBD samples and recent low KBD incidence rate, most previous KBD studies have focused on adult KBD, which provides limited information regarding the mechanism of KBD cartilage lesions at an early stage.

To identify the genes involved in the development of KBD at an early stage, the expression levels of 367 genes in KBD children and adults were evaluated and the differentially expressed genes of each group were identified. 367 KBD genes were selected from results of previous genome-wide gene expression profile analysis in PBMCs and articular cartilage from KBD patients [5]–[7]. Microarray analysis was conducted to evaluate the expression levels of those genes in peripheral blood mononuclear cells (PBMCs) of KBD children and adults respectively. Quantitative real-time reverse transcription polymerase chain reaction (qRT-PCR) was performed to validate the accuracy of the microarray data. In this study, new evidence is presented suggesting that bone development-related genes GDF5 (expression ratio = 2.14±0.02) and DIO2 (expression ratio = 0.11±0.05) are involved in the development of early-stage cartilage lesions in KBD children. This is believed to be the first comparative gene expression analysis between children and adults with KBD.

## Materials and Methods

### Ethics Statement

This study was approved by the Institutional Review Board (IRB) of Xi'an Jiaotong University. All adult subjects or the respective guardians of child subjects gave their informed written consent by signing a document that had been carefully reviewed by the IRB of Xi'an Jiaotong University.

### Sample Collection

All child KBD subjects were randomly chosen from the KBD-endemic area of Tongde County and Guide County in Qinghai Province of China, while adult KBD subjects were chosen from another KBD-endemic area of Yongshou County in Shaanxi Province of China. Healthy subjects came from the same KBD-endemic areas with the KBD case group. All subjects were Chinese Han people. KBD patients were diagnosed strictly according to national diagnostic criteria of Kashin-Beck disease in China [WS/T 207-2010]. Any subjects who had a history of other bone or joint diseases were excluded from this study. Child subjects were divided into six pairs, and adult subjects were divided into seven pairs, each consisting of three KBD patients (1 male and 2 females) and three healthy individuals (1 male and 2 females) matched according to age and gender. Microarray analysis was performed for four of the six child pairs, and qRT-PCR was performed for the remaining two pairs. Likewise, microarray analysis was performed for five of the seven adult pairs, and qRT-PCR was performed for the remaining two pairs. The basic characteristics of the study subjects are shown in Table 1.

**Table 1:** Basic characteristics of the study subjects.

	Adult				Child			
	KBD		Control		KBD		Control	
	n	Age,years(range)	n	Age,years(range)	n	Age,years(range)	n	Age,years(range)
Microarray sample set	3	52.33(50–55)	3	48.67(45–55)	3	12.33(12–13)	3	11.67(11–12)
	3	52.33(48–56)	3	49.67(43–58)	3	12.00(10–13)	3	11.67(10–13)
	3	54.00(48–60)	3	49.67(38–61)	3	12.33(11–13)	3	12.00(11–13)
	3	54.33(45–60)	3	50.00(41–56)	3	11.67(10–13)	3	12.00(10–13)
	3	54.67(40–65)	3	51.67(46–59)	-	-	-	-
qRT-PCR sample set	3	57.67(48–68)	3	49.67(41–57)	3	12.33(12–13)	3	11.67(10–13)
	3	56.67(51–63)	3	50.00(42–55)	3	11.67(9–13)	3	12.33(12–13)
Total	21	54.56(40–68)	21	49.90(38–61)	18	12.05(9–13)	18	11.89(10–13)

### RNA Extraction

Three milliliters of peripheral blood from every child and adult subject was collected and stored at −80°C. Blood samples were thawed at room temperature for 2 hours before RNA extraction. Total RNA was extracted with the Paxgene Blood RNA Kit (PreAnalytix; Qiagen) following the manufacturer's instructions. The integrity of RNA was validated using 1% agarose gel electrophoresis. Extracted RNA was stored at −80°C until cDNA synthesis.

### Selection of Target Genes

**Figure 1:**
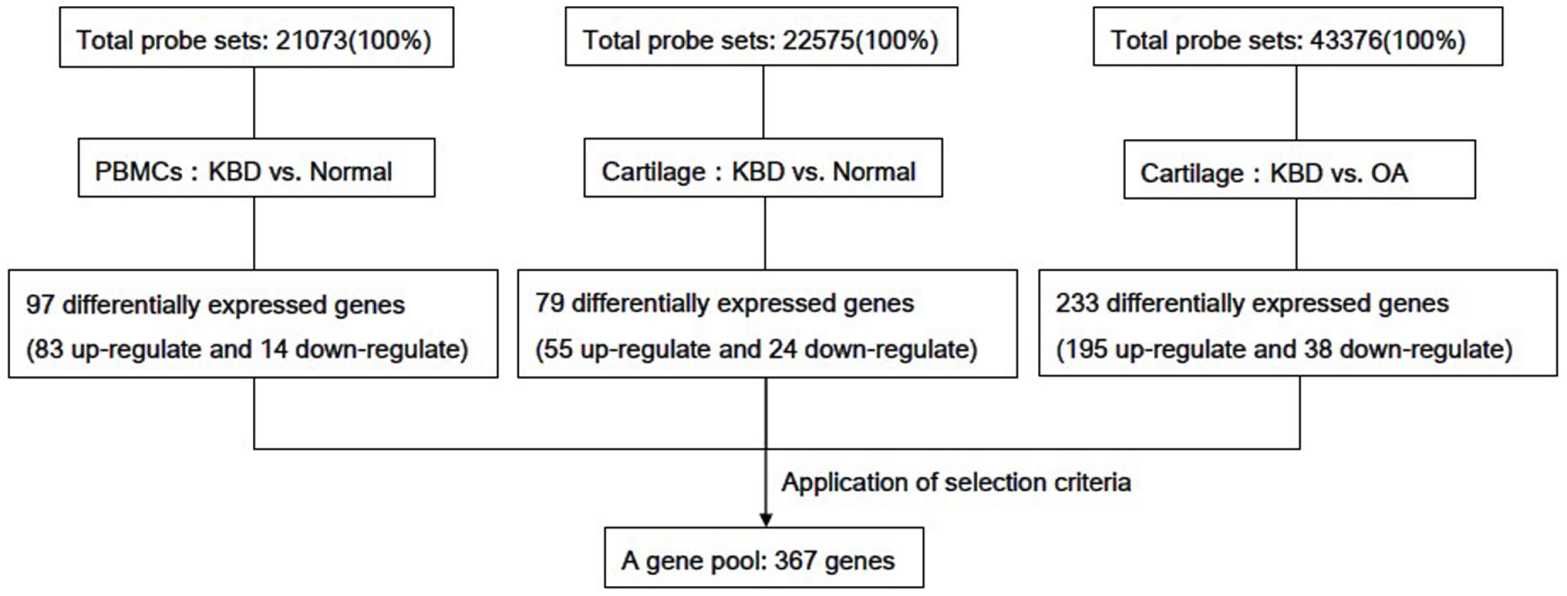
Flow chart of the procedure used to select target genes. This study is based on three additional gene microarray analyses [5]–[7].

In this analysis, 367 genes were selected as target genes from 3 additional databases of previous microarray analysis carried out by other researchers [5]–[7]. In those analyses, a target gene with ≥2 or ≤0.5 fold-change in gene expression level and *P*≤0.05 was regarded as differentially expressed. Overlapping differentially expressed genes and some differentially expressed genes whose function may be not related to cartilage were excluded (detailed in Figure 1).

### Microarray Hybridization

Extracted total RNA was reverse-transcribed into complementary DNA (cDNA), and then transcribed into cRNA and labeled with CyDye with the Amino Allyl MessageAmp aRNA Kit (Ambion) according to the manufacturer's instructions. For each group, 0.5 µg of labeled cRNA was purified separately and then mixed together with hybridization buffer (Agilent In Situ Hybridization Plus kit) before being applied to microarrays. The customized primer array from the National Engineering Research Center for Miniaturized Detection System in China, which contains 367 oligonucleotide probes representing 367 human genes, was used to perform microarray hybridization following the recommended protocol. This is a two-color microarray which uses a two-channel labeling system. The control cRNA was labeled with Cy5 while the KBD cRNA was labeled with Cy3. The array design information has been loaded into the repository of ArrayExpress with the accession number A-MEXP-2399. Hybridization signals were recorded by an Agilent G52565BA scanner, and analyzed using Feature Extraction 9.3 (Agilent Technologies) and Spotfire 8.0 (Spotfire Inc., Cambridge, MA, USA) software. The fluorescent spots that failed to pass the quality control procedure were not used for further analysis. Linear and LOWESS normalization was conducted to eliminate possible dye-related bias in the microarray data. The gene expression data obtained from Spotfire 8.0 was imported into Excel spreadsheets (Microsoft Corp., Redmond, WA, U.S.A.) for downstream data analysis. The dataset of this study has been deposited in ArrayExpress under the accession number E-MTAB-2494.

### Analysis of Microarray Data

To identify differentially expressed genes, expression ratio was calculated for each gene through dividing the fluorescent value of KBD patient by that of control subject. Genes with expression ratios ≤0.5 or ≥2.0 were regarded as differentially expressed in this study. *P* values were calculated using the Agilent *P* value log ratio algorithm [5],
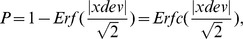
with *Erf(x)* calculated as:
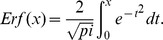

*Erf(x)* represents twice the integral of the Gaussian distribution, with a mean value of 0, variance of 0.5, and *xdev* denotes the deviation of the log ratio from 0. This calculation provides the statistical significance of the log ratio for each feature (*i.e.*, transcript level) between red and green channels. Only *P* values<0.05 were regarded as statistically significant.

### Quantitative Real-Time Reverse Transcription PCR

To validate the microarray results of the adult group, six significant differentially expressed genes were selected for parallel qRT-PCR analysis, including Gpx4 (glutathione peroxidase 4, NM_001039847), CYB5A (cytochrome b5 type A, NM_001914), MGAT3 [mannosyl (beta-1,4-)-glycoprotein beta-1,4-N-acetylglucosaminyltransferase, NM_002409], COL1A1 (collagen, type I, alpha 1, NM_000088), HAPLN1 (hyaluronan and proteoglycan link protein 1, NM_001884) and HBB (hemoglobin, beta, NM_000518). In the validation experiment of the child group, the selected genes are Gpx4, COL1A1, HAPLN1, MGAT3, and HBB. Total RNA was isolated in the same manner as for oligonucleotide array analysis. Extracted total RNA was then converted into cDNA using superscript II reverse transcriptase (Invitrogen, Carlsbad, CA) and random primers. The ABI7500 Real-Time PCR system (Applied Biosystems, Foster City, CA, USA) was used to perform qRT-PCR experiment according to the manufacturer's instructions. All primer and probe sets were purchased from TaqMan Gene Expression Assays (Applied Biosystems). GAPDH (Glyceraldehyde-3-phosphate dehydrogenase, NM_002046) was simultaneously assayed as an endogenous invariant control for data normalization. The expression values of the selected genes were normalized to the expression level of GAPDH. Paired *t* tests were performed to determine significance levels of expression differences for the selected genes between KBD and healthy controls.

## Results

### Clinical and Radiological Characteristics of Representative Children and Adults with KBD

**Figure 2:**
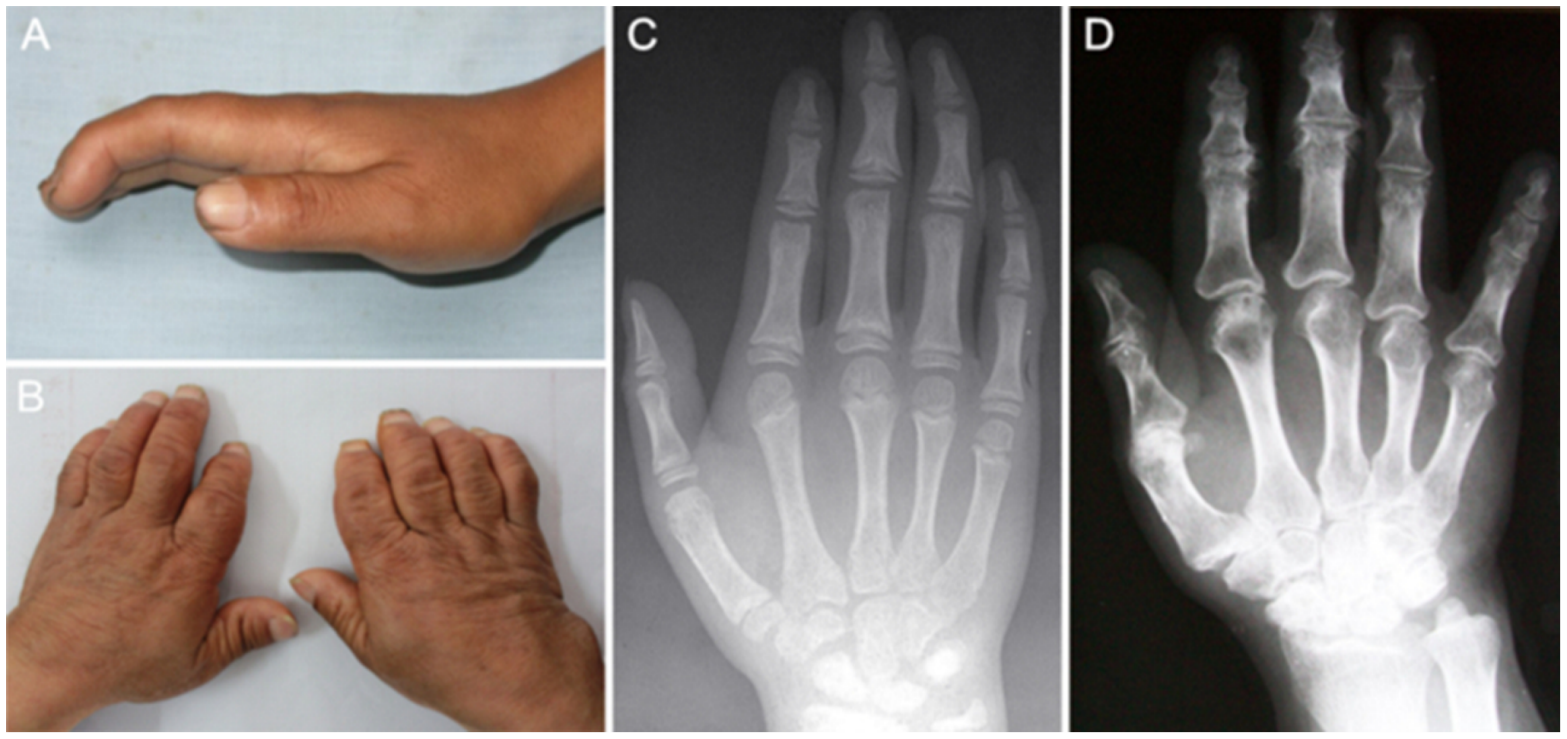
Characteristics of child and adult Kashin-Beck disease (KBD) patients. A, C: Image and radiograph of the left hand of a representative KBD child with grade I KBD (male, 12 years old). B, D: Image and radiograph of hands of a representative KBD adult with grade II KBD (female, 55 years old).

Children and adults with KBD are usually at different stages of KBD and thus experience different typical pathological changes (Figure 2). Flexion of the terminal parts of the finger (Figure 2A) typically emerges earlier than enlarged finger joints. The primary radiographic changes seen in KBD children are metaphyseal lesions in phalanges, including a cone-shaped and blurred margin of the epiphysis, and abnormal closure of the epiphyseal line from the center (Figure 2C). The premature closure of the metaphyseal plate in KBD children results in irregular and impaired development of bones, such as enlarged and crooked joints, as well as shortened fingers (Figure 2B). As seen in radiograph of KBD adults (Figure 2D), extensive articular cartilage damages appear in advanced cases of KBD. The metaphysis has also completely closed and become enlarged, and the margin shows irregular marginal sclerosis. In addition, irregular bone ends, narrowed joint space, and marginal blurred carpal bones are also characteristics for adult KBD patients.

### Microarray Data Analysis

In this study, 95 (25.9%) differentially expressed genes (31 up-regulated and 64 down-regulated) were identified in KBD children, and 158 (43.1%) differentially expressed genes (58 up-regulated and 100 down-regulated) were identified in KBD adults. Further analysis of the differentially expressed genes in KBD children relative to KBD adults revealed that 42 of 95 differentially expressed genes were differentially expressed only in KBD children, but not in KBD adults, including 16 up-regulated genes (Table 2) and 26 down-regulated genes (Table 3). Those 42 genes are involved in various biological processes, such as bone development, growth factors and apoptosis. In addition, 105 differentially expressed genes were differentially expressed only in KBD adults, but not in KBD children, including 46 up-regulated genes and 59 down-regulated genes (Table 4). These 105 genes cover a wild range of biological processes, such as metabolism, apoptosis, immune function and redox system.

**Table 2:** List of genes only differentially expressed in child KBD group (up-regulated genes).

Gene symbol	Gene title	Gene ID	Fold change
			Mean±SEM
*Bone Development*			
GDF5	growth differentiation factor 5	NM_000557	2.14±0.02
*Growth factor*			
PTN	pleiotrophin (heparin binding growth factor 8, neurite growth-promoting factor 1)	NM_002825	2.16±0.86
*Apoptosis*			
RNF34	ring finger protein 34	NM_194271	3.22±0.35
TNFSF11	tumor necrosis factor (ligand) superfamily, member 11	NM_003701	3.21±0.10
*Signal transduction*			
SRP19	signal recognition particle 19 kDa	NM_003135	3.03±0.49
SSBP1	single-stranded DNA binding protein 1	NM_003143	2.31±0.97
SH3GL3	SH3-domain GRB2-like 3	NM_003027	2.33±0.66
*Transcription related*			
LDB1	LIM domain binding 1	NM_003893	2.06±0.45
*Protein synthesis and modification*			
MRPS23	mitochondrial ribosomal protein S23	NM_016070	2.52±0.35
*Ion channel transport protein*			
ENSA	endosulfine alpha	NM_004436	2.06±0.66
*Cytoskeleton and movement*			
TNNT2	troponin T type 2 (cardiac)	NM_000364	2.60±0.31
SYNPO2L	synaptopodin 2-like	NM_024875	2.23±0.61
TNFAIP6	tumor necrosis factor, alpha-induced protein 6	NM_007115	2.21±0.72
*Metabolism*			
CTSC	cathepsin C	NM_001814	2.19±0.42
*Miscellaneous*			
FABP4	fatty acid binding protein 4, adipocyte	W60781	2.35±0.08
FAM104A	family with sequence similarity 104, member A	NM_032598	2.15±0.18

**Table 3:** List of genes only differentially expressed in child KBD group (down-regulated genes).

Gene symbol	Gene title	Gene ID	Fold change
			Mean±SEM
*Bone Development*			
DIO2	deiodinase, iodothyronine, type II	NM_000793	0.11±0.05
*Apoptosis*			
BAX	BCL2-associated X protein	NM_138764	0.31±0.20
*Signal transduction*			
SIGLEC8	sialic acid binding Ig-like lectin 8	NM_014442	0.08±0.02
RASD1	dexamethasone-induced 1	NM_016084	0.22±0.09
GPRC5C	G protein coupled receptor, family C, group 5, member C	NM_022036	0.15±0.01
FLJ41603	FLJ41603 protein	NM_001001669	0.35±0.20
PPM1A	protein phosphatase 1A	NM_021003	0.40±0.19
*Transcription related*			
SCAND2	SCAN domain containing 2	NR_004859	0.10±0.05
FKBP9	FK506 binding protein 9	NM_007270	0.13±0.07
SNAI2	snail family zinc finger 2	U97060	0.19±0.07
EGR1	early growth response 1	NM_001964	0.32±0.11
ZIC5	Zic family member 5 (odd-paired homolog, Drosophila)	NM_033132	0.32±0.05
ZNF562	zinc finger protein 562	NM_017656	0.14±0.03
*Protein synthesis and modification*			
TTC25	tetratricopeptide repeat domain 25	NM_031421	0.11±0.02
DNAJB2	DnaJ (Hsp40) homolog, subfamily B, member 2	NM_001039550	0.15±0.11
*Ion channel transport protein*			
CACNG6	Calcium channel, voltage-dependent, gamma subunit 6	NM_145814	0.17±0.11
*Cytoskeleton and movement*			
EPB49	erythrocyte membrane protein band 4.9 (dematin)	NM_001978	0.26±0.06
TPGS2	tubulin polyglutamylase complex subunit 2	NM_015476	0.45±0.10
*Metabolism*			
PPP3R1	protein phosphatase 3 (formerly 2B), regulatory subunit B, 19 kDa, alpha isoform (calcineurin B, type I)	NM_000945	0.33±0.11
ERH	enhancer of rudimentary homolog (Drosophila)	NM_004450	0.40±0.20
*DNA synthesis and repair*			
REXO2	RNA exonuclease 2 homolog (*S. cerevisiae*)	NM_015523	0.27±0.12
*cytochrome*			
CYP2B6	cytochrome P450, family 2, subfamily B, polypeptide 6	NM_000767	0.19±0.14
*redox related*			
GPX6	Glutathione peroxidase 6 (olfactory)	NM_182701	0.34±0.04
*Miscellaneous*			
ZDHHC2	zinc finger, DHHC-type containing 2	NM_016353	0.28±0.10
SEZ6L2	seizure related 6 homolog (mouse)-like 2	NM_201575	0.29±0.16
FBXO15	F-box protein 15	NM_152676	0.31±0.23

**Table 4:** List of main genes only differentially expressed in adult KBD group.

Gene symbol	Gene title	Gene ID	Fold change
			Mean±SEM
*Apoptosis*			
CASP8AP2	caspase 8 associated protein 2	NM_012115	0.15±0.03
BCL2L1	BCL2-like 1	NM_001191	0.29±0.03
*Immune system function*			
CCL4	chemokine (C-C motif) ligand 4	NM_002984	0.36±0.08
EBI2	EBV-induced G-protein coupled receptor 2	NM_004951	0.39±0.09
HLA-C	Major histocompatibility complex, class I, C	BC002463	2.41±0.31
*Cytoskeleton and movement*			
SGCD	sarcoglycan, delta (35 kDa dystrophin-associated glycoprotein)	NM_000337	0.36±0.01
VIM	vimentin	NM_003380	0.36±0.02
TMOD1	tropomodulin 1	NM_003275	2.65±0.22
GABARAPL2	GABA(A) receptor-associated protein-like 2	NM_007285	2.15±0.37
*Signal transduction*			
RGS12	regulator of G-protein signaling 12	NM_002926	2.14±0.16
GPR20	G protein-coupled receptor 20	NM_005293	3.83±1.34
*Transcription related*			
ATF2	activating transcription factor 2	AK128731	0.29±0.07
ZNF493	zinc finger protein 493	NM_175910	0.43±0.05
*Protein synthesis and modification*			
HSPB9	heat shock protein, alpha-crystallin-related, B9	NM_033194	2.16±0.36
UBB	ubiquitin B	NM_018955	2.04±0.26
*collagen*			
COL5A2	collagen, type V, alpha 2	NM_000393	0.29±0.06
*redox system*			
GPX1	glutathione peroxidase 1	NM_000581	2.28±0.67
GPX2	glutathione peroxidase 2	NM_002083	2.32±0.65
GPX3	glutathione peroxidase 3	NM_002084	2.42±0.48
*Ion transport*			
PLN	phospholamban	NM_002667	0.48±0.06
*Metabolism*			
ACSL6	acyl-CoA synthetase long-chain family member 6	NM_001009185	0.44±0.09
OSBP2	oxysterol binding protein 2	NM_030758	3.20±0.48
*Miscellaneous*			
THBS1	thrombospondin 1	NM_003246	2.64±0.15

Additionally, this study identified 53 differentially expressed genes shared by both KBD children and adults. Sixteen of the 53 genes were found to be expressed asynchronously between KBD children and adults, including 9 genes found to be up-regulated in KBD children but down-regulated in KBD adults, and 7 genes found to be up-regulated in KBD adults but down-regulated in KBD children (detailed in Appendix S1).

### qRT-PCR Validation of Microarray Data

**Figure 3:**
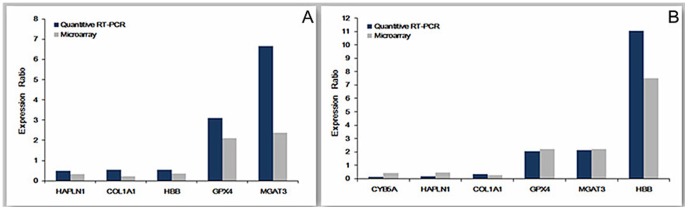
The results of qRT-PCR validation experiment. A: qRT-PCR results of the KBD child group. B: qRT-PCR results of the KBD adult group. Histogram shows the expression ratios of the selected genes.

The results of qRT-PCR experiment were consistent with microarray analysis in both KBD children and adults (Figure 3). According to qRT-PCR results, the expression levels of Gpx4, MGAT3 were higher in children with KBD than healthy children (expression ratio≥2), whereas expression levels of COL1A1, HAPLN1, and HBB were lower in children with KBD than healthy children (expression ratio≤0.5). Expression levels of Gpx4, MGAT3, and HBB were higher in adults with KBD than healthy adults (expression ratio≥2), whereas expression levels of COL1A1, CYB5A, and HAPLN1 were lower in adults with KBD than healthy adults (expression ratio≤0.5).

## Discussion

Gene expression comparative analysis was conducted using customized microarray in KBD children and adults. Significant differences in gene expression pattern were observed between children and adults with KBD. Forty-two genes (16 up-regulated and 26 down-regulated) were found to be differentially expressed in KBD children, but not in KBD adults. Among these genes, bone development-related genes, such as GDF5 (expression ratio = 2.14±0.02) and DIO2 (expression ratio = 0.11±0.05), are of particular importance, and may provide valuable information for further investigation of KBD development at an early stage.

Endochondral bone formation is responsible for most longitudinal bone growth. During this process, chondrocytes in the growth plate undergo a process of maturation and terminal differentiation, which is precisely regulated by a series of signaling molecules. Any disturbances in this complicated regulation system could lead to shortened, or even deformed bones. In the pathology of KBD cartilage, chondronecrosis appears in the mature chondrocytes of the growth plate and articular cartilage [3], which can result in impaired endochondral ossification and skeletal development.

GDF5 is a member of the bone morphogenetic protein (BMP) family. It plays an important role in the development of the skeletal system [8]. A missense mutation of this gene results in chondrodysplasia Grebe-type (CGT), which is an autosomal recessive disorder exhibiting severe limb shortening and dysmorphogenesis [9]. Additionally, a functional polymorphism site of GDF5 is associated with susceptibility to osteoarthritis [10]. Overexpression of a homologue of GDF5 in chickens can increase the length and size of skeletal elements [11]. In humans, it has been shown that over-expression of GDF5 and its receptors is associated with dedifferentiation of human articular chondrocytes, which also can be seen in KBD [12], [13]. The polymorphism of haplotype TGC on GDF5 is found to be significantly associated with KBD, confirming the potential role of GDF5 in the development of KBD [14]. Based on this evidence, it appears that over-expression of GDF5 may play a potential role in the pathology of KBD, although the mechanism needs further evaluation.

The DIO2 gene encodes the deiodinase type 2 (D2), which removes an iodine atom from tetraiodothyronine (T4) to catalyze its conversion into triiodothyronine (T3), the active form of thyroid hormone. Thyroid hormone is a systemic factor that potently regulates skeletal maturation in the growth plate [15]. Two essential trace elements, selenium and iodine, are very important for synthesis and function of DIO2 which is a selenoprotein. Selenium and iodine deficiency are two important environmental factors for KBD [16], [17]. Previous studies have shown that the average level of blood T3 is lower in preschool children from a KBD-endemic country [18]. Additional studies have found that hypothyroidism occurs more frequently in KBD children and adolescents than control subjects [16]. These studies indicate that the interaction of low selenium and iodine levels, thyroid hormone, and DIO2 may contribute to cartilage lesions in KBD children.

Additionally, 105 genes were identified to be differentially expressed in KBD adults, but not KBD children. Among them, 46 genes were up-regulated and 59 genes were down-regulated. We noticed that different members of glutathione peroxidase family were involved in adult KBD and child KBD. For example, GPX1, GPX2, GPX3 were abnormally expressed only in KBD adults (expression ratio = 2.28±0.67, 2.32±0.65, 2.42±0.48, respectively) rather than KBD children. Besides that, GPX4 was up-regulated (expression ratio = 2.23±0.57) and GPX7 was down-regulated (expression ratio = 0.47±0.23) in KBD adults. In KBD children, the involved glutathione peroxidase members were GPX4, GPX6, GPX7 (expression ratio = 2.12±0.43, 0.34±0.04, 2.20±0.86, respectively). KBD is an oxidative stress-related disease [19] and members of the glutathione peroxidase family are responsible for antioxidant function of organism. More glutathione peroxidase members are involved in adult KBD. It may be because oxidative is more severe and extensive in KBD adult.

Some genes related to immune system function were also found to be differentially expressed in KBD adults rather than children, such as HLA-C, CCL4 and EBI2 (expression ratio = 0.36±0.08, 0.39±0.09, 2.41±0.31, respectively). HLA-C encodes the HLA class I heavy chain paralogue. Class I molecules play an important role in the immune system through presenting peptides derived from endoplasmic reticulum lumen. The protein encoded by CCL4 is a mitogen-inducible monokine, which has chemokinetic and inflammatory functions. EBI2 is expressed in B-lymphocyte cell lines but its function is not clear yet. In adult KBD patients, secondary osteoarthritis is the primary clinical manifestation. These genes may be related to the development of secondary osteoarthritis of KBD.

Between children and adults with KBD, 16 common differentially expressed genes were found to be expressed asynchronously (9 up-regulated in KBD children but down-regulated in KBD adults, and 7 up-regulated in KBD adults but down-regulated in KBD children). To study the different roles these genes may play in different stages of KBD and how their expression levels change during development of KBD may be critical to understand KBD and warrants further research.

For example, APAF1, which encodes a cytoplasmic protein that initiates apoptosis, was found to be up-regulated (expression ratio = 2.90±0.30) in KBD children but down-regulated (expression ratio = 0.34±0.04) in KBD adults. The protein product of this gene forms an oligomeric apoptosome in the presence of cytochrome c and dATP, which binds and cleaves caspase 9 preproprotein to make it release its mature, activated form. Activated caspase 9 stimulates the subsequent caspase cascade that commits the cell to undergo apoptosis [20]. Abnormal chondrocyte apoptosis has been proven to appear in the articular cartilage of both children and adults with KBD [21], [22]. Amounts of apoptosis related factors, such as BCL2 and CASP6, among others, as well as related pathways, have been shown to be expressed abnormally in articular cartilage of adult KBD patients [7], [22]–[24]. Additionally, APAF1 was found to be up-regulated in articular cartilage of KBD adults [7]. APAF1 thus appears to be critical for the abnormal apoptosis seen in articular cartilage of KBD patients, although it exerts different biological effects at different stages of KBD.

In summary, the gene expression levels of children and adults with KBD were compared, and significant differences in gene expression pattern were found between KBD children and adults, which may indicate the molecular mechanism underlying cartilage lesions. In addition, the results of this study revealed several genes that are only abnormally expressed in KBD children or KBD adults, as well as some common differentially expressed genes that are expressed asynchronously between KBD children and adults. The study results suggest that bone development-related genes GDF5 and DIO2 may play a potential role in the development of KBD in children rather than in adults and may provide a basis for further study of the molecular mechanism of KBD, particularly in children.

## Supporting Information

Appendix S1 **List of common differentially expressed genes in two groups.** Although these differentially expressed genes were shared by two groups, they expressed asynchronously in KBD children and KBD adults. This table includes 7 genes down-regulated in KBD children but up-regulated in KBD adults and 9 genes down-regulated in KBD adults but up-regulated in KBD children.
(DOC)
