# Supplementary material for: Gene Expression Analysis Suggests Bone Development-Related Genes GDF5 and DIO2 Are Involved in the Development of Kashin-Beck Disease in Children Rather than Adults
Source: PLoS One. 2014 Jul 29;9(7):e103618. doi: 10.1371/journal.pone.0103618 (PMC4114804; doi:10.1371/journal.pone.0103618)
Supplement: Appendix S1 — List of common differentially expressed genes in two groups. Although these differentially expressed genes were shared by two groups, they expressed asynchronously in KBD children and KBD adults. This table includes 7 genes down-regulated in KBD children but up-regulated in KBD adults and 9 genes down-regulated in KBD adults but up-regulated in KBD children. (DOC) [file pone.0103618.s001.doc]

| **Gene symbol** | **Gene Title** | **Gene ID** | **Fold change** | |
| --- | --- | --- | --- | --- |
|  |  |  | **Mean±SEM** | |
|  |  |  | **Children** | **Adults** |
| *Genes down-regulated in KBD children but up-regulated in KBD adults* | | | | |
| WNK1 | WNK lysine deficient protein kinase 1 | NM_018979 | 0.08±0.02 | 3.69±1.50 |
| SCNN1B | sodium channel, nonvoltage-gated 1,beta | NM_000336 | 0.16±0.05 | 2.84±1.01 |
| RSRC1 | arginine/serine-rich coiled-coil 1 | NM_016625 | 0.21±0.07 | 4.05±3.81 |
| SEC14L4 | SEC14-like 4 (S. cerevisiae) | NM_174977 | 0.23±0.11 | 2.05±0.57 |
| ROBO3 | roundabout,axon guidance receptor, homolog 3 (Drosophila) | NM_022370 | 0.30±0.37 | 2.17±0.68 |
| MPP1 | membrane protein, palmitoylated 1 | NM_002436 | 0.36±0.22 | 2.21±0.49 |
| HBB | hemoglobin, beta | NM_000518 | 0.35±0.04 | 7.51±4.72 |
| *Genes down-regulated in KBD adults but up-regulated in KBD children* | | | | |
| CD3G | CD3g molecule, gamma (CD3-TCR complex) | NM_000073 | 2.16±0.69 | 0.39±0.17 |
| DSTN | destrin (actin depolymerizing factor) | NM_006870 | 2.19±0.81 | 0.33±0.21 |
| GPX7 | glutathione peroxidase 7 | NM_015696 | 2.20±0.86 | 0.47±0.23 |
| BIRC3 | baculoviral IAP repeatcontaining 3 | NM_001165 | 2.27±0.96 | 0.26±0.08 |
| B3GNT1 | UDP-GlcNAc:betaGal beta-1,3-N-acetylglucosam nyltransferase 1 | NM_006577 | 2.45±0.46 | 0.40±0.15 |
| BDP1 | B double prime 1, subunit of RNA polymerase III transcription initiation factor IIIB | NM_018429 | 2.57±0.42 | 0.46±0.14 |
| FBLN1 | fibulin 1 | NM_006486 | 2.71±0.64 | 0.47±0.10 |
| APAF1 | apoptotic peptidase activating factor | NM_181861 | 2.90±0.30 | 0.34±0.04 |
| CASP6 | Caspase 6,apoptosis-relate cysteine peptidase | NM_001226 | 3.45±0.19 | 0.30±0.13 |
